# A next generation sequencing-based method to study the intra-host genetic diversity of norovirus in patients with acute and chronic infection

**DOI:** 10.1186/s12864-016-2831-y

**Published:** 2016-07-01

**Authors:** Maria E. Hasing, Bart Hazes, Bonita E. Lee, Jutta K. Preiksaitis, Xiaoli L. Pang

**Affiliations:** Department of Laboratory Medicine and Pathology, University of Alberta, Edmonton, AB T6G 2B7 Canada; Department of Medical Microbiology and Immunology, University of Alberta, Edmonton, AB T6G 2E1 Canada; Department of Pediatrics, University of Alberta, Edmonton, AB T6G 1C9 Canada; Department of Medicine, University of Alberta, Edmonton, AB T6G 2B7 Canada; Provincial Laboratory for Public Health (ProvLab), Edmonton, AB T6G 2 J2 Canada

**Keywords:** Norovirus, Molecular evolution, Genetic variation, Immunocompromised host, Gastroenteritis outbreaks, Next generation sequencing

## Abstract

**Background:**

Immunocompromised individuals with chronic norovirus (NoV) infection and elderly patients are hypothesized to be reservoirs where NoV might accumulate mutations and evolve into pandemic strains. Next generation sequencing (NGS) methods can monitor the intra-host diversity of NoV and its evolution but low abundance of viral RNA results in sub-optimal efficiency. In this study, we: 1) established a next generation sequencing-based method for NoV using bacterial rRNA depletion as a viral RNA enrichment strategy, and 2) measured the intra-host genetic diversity of NoV in specimens of patients with acute NoV infection (*n* = 4) and in longitudinal specimens of an immunocompromised patient with chronic NoV infection (*n* = 2).

**Results:**

A single Illumina MiSeq dataset resulted in near full-length genome sequences for 5 out of 6 multiplexed samples. Experimental depletion of bacterial rRNA in stool RNA provided up to 1.9 % of NoV reads. The intra-host viral population in patients with acute NoV infection was homogenous and no single nucleotide variants (SNVs) were detected. In contrast, the NoV population from the immunocompromised patient was highly diverse and accumulated SNVs over time (51 SNVs in the first sample and 122 SNVs in the second sample collected 4 months later). The percentages of SNVs causing non-synonymous mutations were 27.5 % and 20.5 % for the first and second samples, respectively. The majority of non-synonymous mutations occurred, in increasing order of frequency, in p22, the major capsid (VP1) and minor capsid (VP2) genes.

**Conclusions:**

The results provide data useful for the selection and improvement of NoV RNA enrichment strategies for NGS. Whole genome analysis using next generation sequencing confirmed that the within-host population of NoV in an immunocompromised individual with chronic NoV infection was more diverse compared to that in individuals with acute infection. We also observed an accumulation of non-synonymous mutations at the minor capsid gene that has not been reported in previous studies and might have a role in NoV adaptation.

**Electronic supplementary material:**

The online version of this article (doi:10.1186/s12864-016-2831-y) contains supplementary material, which is available to authorized users.

## Background

Norovirus (NoV) is recognized as a leading cause of epidemic and sporadic gastroenteritis around the world [1]. The viral RNA genome is about 7500 nt long and contains three ORFs. ORF1 encodes for a polyprotein that is cleaved into 6 non-structural proteins [2]. ORF2 and ORF3 encode the major (VP1) and minor (VP2) capsid proteins, respectively. NoVs are classified, based on VP1 amino acid sequences, into seven genogroups (GI-GVII) that are further divided into genotypes. To date, 41 NoV genotypes have been reported of which at least 29 have been found in humans [3], however, genotype GII.4 alone is responsible for over 60 % of all NoV outbreaks worldwide [4]. New genetic clusters of GII.4, commonly referred to as GII.4 variants, arise every 2 to 4 years and spread rapidly often causing global pandemics [4]. Novel GII.4 variants evolve by antigenic drift and display changes in VP1 epitopes that can avert immune responses mounted against previous variants [5–7]. Homologous recombination is another mechanism responsible for the genetic diversity among NoV GII.4 and it commonly occurs at the ORF1/ORF2 junction allowing the virus to exchange structural and nonstructural genes between different GII.4 variants or even different genotypes [8, 9].

Norovirus acute gastroenteritis is a self-limited illness typically lasting 2 to 3 days while viral shedding can range from 13 to 56 days [10]. In immunocompromised patients, however, NoV shedding is usually prolonged [11–15] and cases of chronic NoV infection with shedding over 1–2 years have been reported [13, 14, 16]. Due to their long shedding periods and weak immune responses it has been hypothesized that immunocompromised patients with chronic NoV infection might be reservoirs where new GII.4 variants emerge [12, 17]. Indeed, ORF2 sequence analysis in these individuals has shown that the virus can accumulate mutations in VP1 and develop an intra-host NoV population with large genetic diversity [12, 16, 18–23]. However, it is still unclear how other regions of the viral genome evolve in immunocompromised hosts. More recently, the elderly and malnourished host have also been proposed as NoV reservoirs where NoV might accumulate mutations [17] but no studies have yet been performed in humans to confirm this hypothesis.

The gold standard method to study intra-host NoV populations involves cloning viral RT-PCR amplicons into plasmids followed by Sanger sequencing [16, 19, 20, 24], a labour intensive method that requires a relatively large number of clones to be processed in order to obtain an accurate assessment of the viral diversity. An alternative approach uses next generation sequencing (NGS) technologies, which process millions of fragments of nucleic acid in a single experiment. NGS is highly cost-effective in terms of cost per base, however, since NoV RNA represents a very small fraction of all stool RNA, as little as 0.01 % [25], the cost per viral base can be considerable. Fortunately, the efficiency can be improved by viral RNA enrichment, and strategies previously used for NoV enrichment include polyA tail selection [26], RT-PCR amplification using NoV-specific primers [21, 23, 27], VIDISCA [28, 29] and virus purification [22]. Depletion of rRNA is another possible enrichment strategy [30]. For most cells, rRNA is the most abundant species of RNA and its removal with commercial kits can substantially increase the prevalence of non-rRNA [31, 32]. The method poses an advantage over others in that it maintains the original representation of the viral population present in the sample and is less likely to be affected by NoV RNA fragmentation.

In this study we establish a next generation sequencing-based method for NoV using bacterial rRNA depletion as an enrichment strategy for NoV RNA then use the resulting data to examine the intra-host viral population in samples from patients involved in NoV outbreaks (mostly elderly) and in longitudinal samples collected from an immunocompromised host with chronic NoV infection.

## Results

### Mapping of NoV sequencing reads

**Fig. 1:**
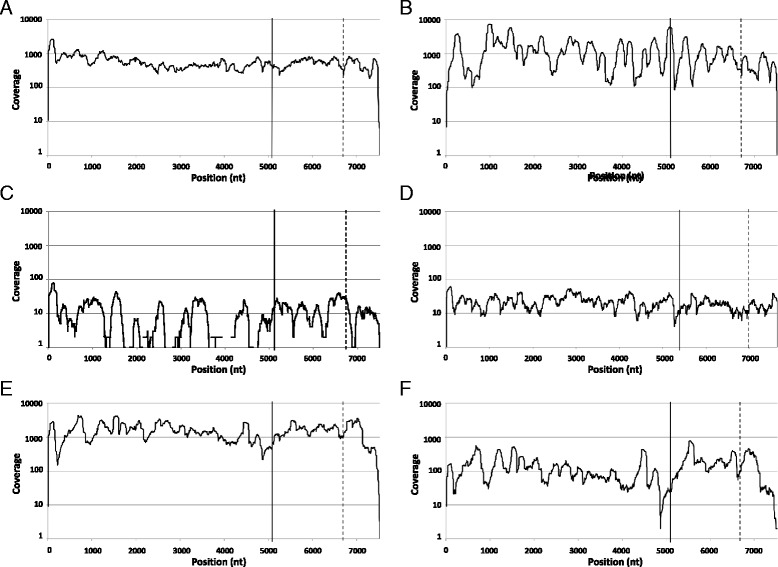
Coverage per NoV genome position. Number reads (in log scale) that aligned with the corresponding NoV consensus sequence of OU1 (**a**), OU2 (**b**), OU3 (**c**), OU4 (**d**), SP1 (**e**) and SP2 (**f**). Solid and broken lines indicate the start of ORF2 and ORF3, respectively

The samples included in this study (*n* = 6) are described in Table 1. NoV sequences represented 0.01 % to 1.88 % of all quality-filtered reads and 0.04 % to 8.54 % of the non-rRNA reads. The non-NoV reads were further characterized and a description is provided under Additional file 1. The average coverage of the final consensus sequences ranged between 11X and 1,603X (Table 2 and Fig. 1). Five out of six samples yielded near full-length NoV sequences and called 99.91 to 100 % of the reference genome. OU3, the sample with the lowest percentage of NoV reads and lowest coverage, failed to yield a complete NoV genome sequence. The percentage of NoV sequences per sample showed a strong correlation with the Ct values of the RT-qPCR performed on RNA stool extracts (Spearman’s rho correlation coefficient: 0.886, *P* = 0.019, two-tailed). An equally strong correlation was observed between the percentage of NoV sequences and viral titer per ng of RNA (Additional file 2). These results suggest that the poor yield of NoV sequences from sample OU3 was due to a low abundance of viral RNA rather than failure of the enrichment method.

**Table 1:** Samples analyzed by NGS

Sample	NoV genotype	Sample collection Date	Patient description, age group
OU1	GII.4 Sydney 2012	January 2012	Outbreak patient (senior residence), 70-90Y
OU2	GII.4 Sydney 2012	September 2014	Outbreak patient (supportive living), > 70-90Y
OU3	GII.5	November 2013	Outbreak patient (hospital acute care), 30-50Y
OU4	GI.7	November 2012	Outbreak patient (senior residence), 70-90Y
SP1	GII.4 Den Haag 2006b	December 2012	Bone marrow transplant patient, < 18Y
SP2	GII.4 Den Haag 2006b	April 2013

**Table 2:** Norovirus reads and coverage per sample

Sample	Reads mapping to NoV	Percentage of NoV reads		Average coverage	% of positions called^a^	Length of consensus sequence (bp)
		Vs. non-rRNA reads	Vs. quality filtered reads			
OU1	37863	4.01 %	0.51 %	590X	100 %	7532
OU2	88270	5.36 %	1.88 %	1318X	99.55 %	7525
OU3	746	0.04 %	0.01 %	11X	90.59 %	N.A.^b^
OU4	1420	2.43 %	0.02 %	22X	N.A.	7657
SP1	102805	8.54 %	1.78 %	1603X	99.91 %	7524
SP2	9589	7.55 %	0.33 %	149X	99.91 %	7535

N.A.: not applicable

^a^Coverage was calculated compared to KF509946 (OU1), KC631827 (OU2), KJ196277 (OU3), JN899243.1 (OU4) and KC576909 (SP1 and SP2)

^b^The alignment of OU3 produced 9 non overlapping contigs of 1744, 281, 518, 394, 434, 106, 119, 487 and 2760 nt long

In order to validate our NGS method, the NoV consensus sequences of samples OU1 and OU3 obtained with the MiSeq platform were compared to those obtained using Sanger sequencing. There was a large concordance between both methods. NoV OU1, which achieved high coverage with MiSeq, showed a concordance of almost 100 % between the sequences from both methods except for one nucleotide. The base mismatch was located near the 3’end of the genome and was confirmed as an error in Sanger sequencing after review of the Sanger chromatogram and the coverage with MiSeq at that position (21X). Furthermore, the 5’ and 3’ ends of the genome could be extended by 9 and 24 nucleotides, respectively, with MiSeq. These nucleotides were not covered with Sanger because of their close proximity to the primers used for PCR amplification and sequencing. The consensus sequence with lowest coverage, NoV OU3, showed three mismatches over a total of 5,081 nt that could be sequenced using Sanger and MiSeq. The mismatches were identified at 1732 nt, 2167 nt and 2239 nt (positions are given relative to KU311160) and had, respectively, coverages of 1X, 2X and 2X and Phred base quality scores of 16, 39 and 39. All three mismatches occurred at wobble bases resulting in synonymous mutations and the last two were located in the p22 gene. Since these positions had very low coverage, it is possible that the mismatches were created by sequencing errors with MiSeq, however, the high base quality associated with at least 2 of these three nucleotides and their relative position in the genome suggests that they could also represent true SNVs originally present in the sample.

### Detection of single nucleotide polymorphisms

**Fig. 2:**
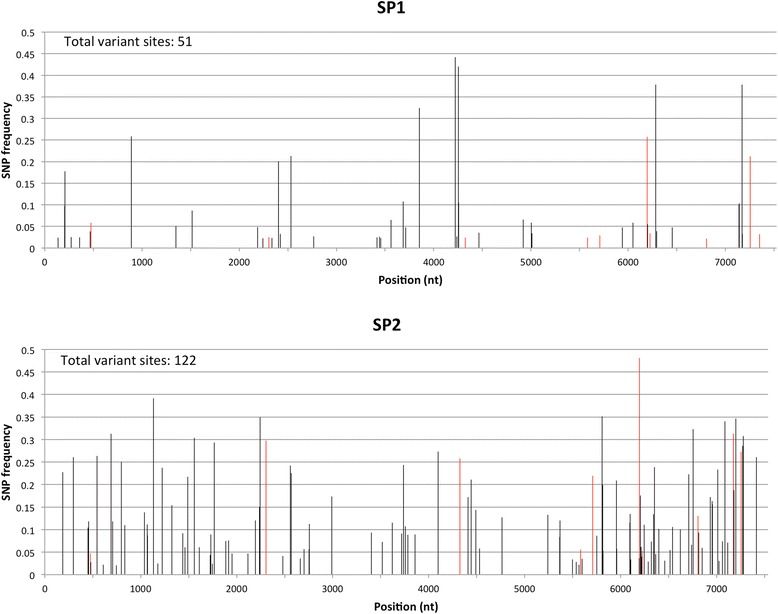
Distribution of NoV SNV frequencies across the viral genome. Samples SP1 and SP2 were collected four months apart from an immunocompromised bone marrow transplant patient with chronic NoV infection. SNV calling was performed using Freebayes. Only SNVs with frequencies **≥ **2 % and **≥ **5X coverage are reported. Positions with coverage < 10X were excluded from the analysis. SNVs shared in common between SP1 and SP2 are shown in red

Single nucleotide variants (SNV) were identified to measure the intra-host genetic population of NoV. In order to reduce false positives, we only called a SNV if it was observed at least 5 times and represented a minimum of 2 % of all observations. Using these criteria we did not detect any SNVs in all acute infection samples (OU1, OU2, OU3 and OU4). OU3 and OU4 have relatively low coverage (Table 2) limiting the ability to detect SNVs, but OU1 and OU2 have an average coverage above 250X and still no SNVs were found. In contrast, the first sample from the immunocompromised subject with chronic NoV infection, SP1, had 51 SNVs while SP2, the second sample collected from the same individual 4 months later, had 122 SNVs, indicating that the genetic diversity of NoV in this patient was higher and also increased over time (Fig. 2). The increased number of SNVs in SP2 vs. SP1 was confirmed even after controlling for differences in coverage (Additional file 3). The higher number of SNVs in the SP2 sample was not due to more sensitive detection, since SP2 had a lower average coverage than SP1, OU1, and OU2 (Table 2).

**Fig. 3:**
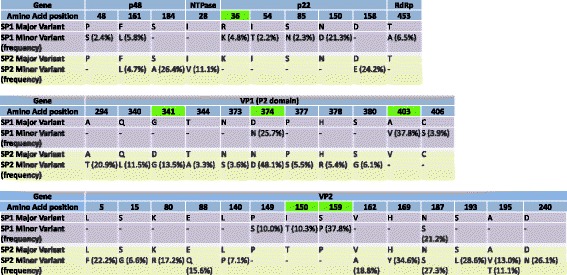
Distribution per gene of the amino acid residues affected by non-synonymous single nucleotide variants in NoV. Samples SP1 and SP2 were collected four months apart from an immunocompromised bone marrow transplant patient with chronic NoV infection. Residues that changed between SP1 and SP2 are highlighted in green

The percentages of non-synonymous single nucleotide variants (nsSNVs) for SP1 and SP2 were 27.5 % and 20.5 %, respectively. There were differences in the distribution of nsSNV across genes between SP1 and SP2. In SP1, most nsSNVs occurred in p22 (4/14), VP2 (4/14) and VP1 (3/14) whereas in SP2, the majority of nsSNVs occurred in VP2 (12/25) and VP1 (9/25) (Table 3). The enrichment of non-synonymous mutations in VP2, the P2 domain of VP1 and p22 was statistically significant (*P* < 0.05) after controlling for gene/domain size (Table 3) and is consistent with an increased rate of amino acid divergence in these proteins. The amino acid residues affected by nsSNVs (summarized in Fig. 3) were mostly scattered across VP2 (5, 15, 80, 88, 140, 149, 150, 159, 162, 169, 187, 193, 195 and 240) whereas for VP1 all were located within the P2 domain (294, 340, 341, 344, 373, 374, 377, 378, 380, 403 and 406). Of the 11 mutations occurring at the P2 domain of VP1, two (affecting amino acid residues 294 and 340) were located at epitopes reported to be targeted by antibodies that block the binding of NoV to human blood group antigens (surrogates of neutralizing antibodies) [6].

**Table 3:** Distribution of single-nucleotide variants in NoV by gene. Single nucleotide variants (SNVs) were identified using FreeBayes in two samples (SP1 and SP2) collected four months apart from an immunocompromised bone marrow transplant patient with chronic NoV infection. Only those SNVs found at frequencies **≥ **2 % and with 5X coverage are reported. Positions with coverage < 10X were excluded from the analysis

ORF	Gene (other names)	Position (nt)	SP1		SP2	
			S	NS (*P*-value*)	S	NS (*P*-value*)
1	p48 (NS1-2, N-term, p37)	1–983	6	2 (0.5)	11	2 (0.8)
	NTPase (NS3)	984–2081	2	0 (1)	18	1 (1)
	p22 (p20, NS4)	2082–2618	3	4 (0.01)	7	1 (0.8)
	VPg (NS5)	2619–3014	1	0 (1)	6	0 (1)
	3CLpro (NS6)	3015–3560	4	0 (1)	2	0 (1)
	RdRp (NS7)	3561–5090	11	1 (0.9)	13	0 (1)
2	VP1	5074–6690	7	3 (0.6)	30	9 (0.06)
	S domain	5074–5736	2	0 (1)	9	0 (1)
	P1 subdomain	5737–5895, 6325–6690	1	0 (1)	13	0 (1)
	P2 subdomain	5896–6324	4	3 (0.03)	8	9 (4x10^-6^)
3	VP2	6693–7496	3	4 (0.04)	10	12 (1x10^-6^)
	TOTAL		37	14	97	25

*P-values were calculated using binomial distribution and indicate the probability of observing the corresponding number of non-synonymous single nucleotide variants in the specified region after controlling for gene/domain size

A comparison between the consensus sequences of SP1 and SP2 revealed differences in 9 positions, of which 2 were synonymous (203 nt and 3851 nt), 6 were non synonymous (2188 nt, 6095 nt, 6193 nt, 6281 nt, 7141 nt and 7167 nt; the corresponding amino acid changes are shown in Fig. 3) and 1 was located at the 3’UTR (7509 nt). Among the 9 differences between the consensus sequences of SP1 and SP2, 6 were SNVs at SP1 only (203 nt, 2188 nt, 3851 nt, 6281 nt, 7141 nt and 7167 nt), 1 was a SNV in SP2 only (6095 nt), 1 was a SNV in SP1 and SP2 (6193 nt) and 1 was not a SNV in SP1 nor SP2 (7509 nt). There were a total of 10 SNVs occurring at the same nucleotide positions of SP1 and SP2 (474 nt, 2303 nt, 4323 nt, 5580 nt, 5706 nt, 6193 nt, 6222 nt, 6803 nt, 7172 nt and 7252 nt; shown with red color in Fig. 2).

## Discussion

In this study we established a method to analyze the intra-host genetic diversity of NoV in samples from patients with acute and chronic NoV infection. Our next generation sequencing-based method coupled to bacterial rRNA depletion as a NoV RNA enrichment strategy produced between 0.01 % and 1.9 % of NoV reads. Characterization of the non-NoV reads confirmed that samples still contained 64 to 95 % of bacterial rRNA reads after depletion which differs with a previous study reporting 1 to 5 % of bacterial rRNA reads after using the same depletion method with stool RNA [32]. The reasons for the lower rRNA depletion efficiency observed with our samples could not be identified. Since the method we used for depletion works with a library of probes that must first hybridize the bacterial rRNA for subsequent removal, it is possible that the hybridization step was affected by sequence incompatibility. Also, rRNA depletion was carried out with the maximum amount of input RNA recommended by the manufacturer, which could have affected the efficiency of the method. Based on our data we estimate that if all 16S and 23S bacterial rRNA could be removed from stool RNA, NoV sequences would have represented between 0.04 % and 8.5 % of all NGS reads.

For all but one sample we were able to obtain sufficient sequence data to retrieve near full-length NoV genome sequences. Moreover, even the non-enriched sample (OU4) provided enough data for *de novo* assembly of a NoV genome, AlbertaEI404/2012/CA, which to our knowledge, is the first near full-length NoV GI.7 genome reported. Strains of genotype GI.7 were observed with increased prevalence in Alberta, Canada, between July 2012 and June 2013 [33] and also among children in Pakistan between April 2006 and March 2008 [34].

Our analysis included four cases of acute NoV infection. Two samples from these acute cases, OU1 and OU2, yielded sufficient coverage to identify SNVs at frequencies ≥ 2 %. However, no NoV SNVs were detected in these two samples, indicating that the viral population in typical acute infections is homogeneous. In contrast, we identified numerous SNVs in an immunocompromised patient with chronic NoV infection. Interestingly, there was also an increase of SNV over time (51 SNVs with ≥ 2 % frequency in the first sample and 122 SNVs with ≥ 2 % frequency in the second sample collected 4 months after), revealing intra-host NoV evolution. As of the date of writing this manuscript there were two published studies comparing the intra-host populations of NoV between immunocompetent and immunocompromised patients by using NGS data. Both reported similar observations [21, 22]. Vega et al. found no SNVs throughout the viral genome (ORF1, ORF2 and ORF3) in an immunocompetent subject with acute NoV illness but identified multiple SNVs in three different immunocompromised bone marrow transplant patients (15, 67 and 235 SNVs with ≥ 10 % frequency). In an analysis of just the ORF2 and partial ORF3 regions, Bull et al. detected multiple SNVs in immunocompetent individuals with acute NoV infection (5 to 8 SNVs with ≥ 2 % frequency). However, an immunocompromised individual with chronic NoV infection displayed considerably higher NoV diversity that also increased over time (48, 59 and 109 SNVs with ≥ 2 % frequency, in three samples collected longitudinally).

Interestingly, about half of the SNVs reported by Bull et al. were non synonymous (46 to 64 % in immunocompetent and 34 to 48 % in immunocompromised subjects) whereas we observed a rate of 21 and 28 % in the immunocompromised patient. Our rates are similar to those reported in another study with bone marrow transplant patients receiving immunosuppressive drugs (236 non synonymous mutations out of a total of 1082 mutations across 13 samples, equivalent to an overall rate of 21.8 %) [23]. It could be argued that Bull et al. achieved higher coverage and therefore higher resolution of SNVs with PCR, however, at least two of our samples (one from an outbreak patient and another from the immunocompromised patient) had average coverage levels greater than 1300X, well above 950X, the average coverage reported by Bull et al. We can only speculate that the differences between our study and that of Bull et al. could be due to the processes of quality filtering and trimming of reads as well as the parameters used for SNV calling, which probably were more stringent in our study.

We observed an enrichment of non-synonymous SNVs in the P2 domain of VP1 which matches the expectation that the most exposed and possibly the most antigenic part of the virus bears the highest pressure to diverge. In fact, the majority of studies analyzing NoV intra-host genetic diversity have been restricted to identify potential changes at VP1 [12, 14, 21, 24, 35, 36]. By analyzing near-full length NoV genomes, we also observed an enrichment of non-synonymous mutations in VP2, which suggests that during chronic infection this gene can also be under pressure to diverge. Although our observations are based on samples from a single immunocompromised patient, we believe this is plausible because it agrees with previous analysis of sequence alignments of multiple GII.4 strains showing high evolutionary rates for VP2 [27, 37]. The role of VP2 in the viral life cycle is still unknown. Only a few copies of VP2 molecules are present in the final virion (the precise number is yet unclear). VP2 appears to bind the interior surface of the capsid and has been shown to enhance the expression and half-life of VP1 [38]. Since VP2 is rich in basic residues and therefore is positively charged, it has also been suggested that it interacts with the negatively charged genomic RNA, and possibly plays a role during the encapsidation process [38]. The VP2 protein of murine noroviruses regulates the maturation of antigen presenting cells and is an important determinant of NoV protective immunity [39]. It is interesting to consider that human NoV VP2 might be an important epitope of host immune responses.

By analyzing whole NoV genome sequences using traditional cloning, Chan et al., found accumulation of mutations at VP1 and VP2 (38 and 15 nt resulting in 9 and 5 amino acid changes, respectively) over a period of 4 months in an immunocompromised patient with agammaglobulinemia and thymoma [19]. Conversely, Vega et al. reported that, in comparison to NoV strains in circulation, NoV mutations in immunocompromised bone marrow transplant patients occur mostly at random positions except for gene p22, which presents a significantly large proportion of mutations and positively selected sites [22]. We speculate that the difference in results observed in these studies and our study might be due to temporal changes in the strength and specificity of host immune responses during chronic infection. This will be dependent on the type of immunosuppressed host, the type of exogenous immunosuppression administered, the time of infection during the patient’s clinical course and the time of the patient’s immune reconstitution. Regarding this point, we observed that the distribution of non synonymous mutations across genes changed with time which suggests that NoV might face changes in its evolutionary trajectory during chronic infection as reported for other viruses such as HCV and HIV [40–42].

Besides whole genome sequencing, NGS methods are powerful tools for studying viral population dynamics within a host because of the high sequencing depth that can be achieved. In addition, samples can be prepared relatively quickly without the need of cloning into vectors and multiple isolates can be sequenced in parallel (in our case, six samples were analyzed in one MiSeq 2x121bp run). The number of samples that can be sequenced depends on the throughput of the sequencing technology and the abundance of the viral RNA in the sample. Different forms of enrichment can enhance the later. We demonstrated that bacterial rRNA depletion is a beneficial treatment that can be further optimized. Hybrid capture is a promising new enrichment strategy that remains to be examined in the future [43].

## Conclusions

This study provides data useful for the selection and improvement of NoV RNA enrichment strategies for NGS and is also the first study to look at the intra-host diversity of NoV by analyzing near full length-genomes from acute cases of NoV infection and in longitudinally collected samples from an immunocompromised patient with chronic NoV infection. We identified a larger viral diversity in the immunocompromised patient, which increased over time and we also observed that genes VP2, p22 and VP1 accumulated the majority of non-synonymous mutations during chronic infection. Further studies are needed to observe if the accumulation of mutations at VP2 is consistent across immunocompromised patients with chronic infection and unveil the role of VP2 and p22 proteins in relationship to host immune responses. We are currently planning a larger follow-up study to assess the intra–host genetic diversity of NoV in solid organ transplant recipients.

## Methods

### Patient samples

All stool specimens were collected in Alberta between 2012 and 2014 and stored at -80 °C until analysis. A total of six stool samples previously genotyped within our routine program of NoV surveillance in Alberta were included in this study [33, 44]. The patient’s description and NoV genotype associated to each sample are listed in Table 1. Samples OU1, OU2, OU3 and OU4 were collected from outbreak patients with acute NoV infection. The near full-length norovirus genome for sample OU1 was determined in a previous study using Sanger sequencing [9] and was included for comparison purposes. Samples SP1 and SP2 were collected four months apart from a pediatric bone marrow transplant patient who first tested positive for NoV 6 months before sample SP1 was collected.

### RNA extraction

Samples OU1, OU2, OU3, SP1 and SP2 were processed as follows: 50 to 75 mg of stool were mixed with 20 μL of proteinase K and 200 μL of lysis buffer from Magazorb®RNA mini-prep kit (Promega, Madison, WI). Nucleic acids were extracted with 1 mL of Trizol® (Life Technologies, Carlsbad, CA) and according to the manufacturer’s instructions. The yield of RNA per extraction was estimated using NanoDrop 1000 and the process was repeated to obtain at least 50ug of RNA per sample. The extracts were then treated with DNase (Promega, Madison, WI) and purified by phenol chloroform extraction and ethanol precipitation. RNA extracts were eluted through OneStep™ PCR Inhibitor Removal columns (Zymo Research, Irvine, CA). Bacterial rRNA was depleted using Ribo-Zero® bacterial kit (Epicentre, Madison, WI) according to the manufacturer’s instructions using 5 μg of RNA per sample and purifying by ethanol precipitation as the final step. Depletion of bacterial rRNA was performed once per sample. For comparison purposes, sample OU4 was processed using our routine enteric virus nucleic acid extraction method previously described [33, 44] using Magazorb®RNA mini-prep kit. The nucleic extract was treated with DNase and purified by phenol chloroform extraction followed by ethanol precipitation. Sample OU4 was not depleted of bacterial rRNA. The presence of norovirus RNA was confirmed in all extracts by RT-qPCR as previously described [45].

### Illumina library preparation and sequencing

Sample libraries were prepared from 1 μg of RNA using the TruSeq RNA sample preparation kit v2 (Illumina, San Diego, CA) following the manufacturer’s instructions with a fragmentation time of 1 min during the “elute-fragment-prime” step and unique indexed adapters for each sample. cDNA libraries were quantified with Qubit and the average fragment size was estimated using the Agilent 2100 Bioanalyzer. A control library of phage X714 was also included in each sample. All sample libraries (*n* = 6) were sequenced once on a single Illumina MiSeq run to produce paired end reads of 250 bp each, resulting in reads of 121 bp each after removing adapters.

### Identification, characterization and removal of rRNA reads

Raw sequence reads were quality-trimmed and filtered with Prinseq-lite, version 0.20.4 [46] using the following criteria: the first nucleotide at each end (5’ and 3’) was trimmed and the following nucleotides were also trimmed stepwise if their base quality was below 20. Sequences with an average base quality below 20 or with more than 90 % of Ns were also removed. A description of the reads that were filtered is provided in Additional file 2.

Ribosomal RNA reads were identified and filtered out with SortmeRNA [47] using the 23S/28S large subunit (LSU) and 16S/18S small subunit (SSU) rRNA SILVA 119 databases and the 5S and 5.8S rRNA Rfam databases for all three domains of life (Eukarya, Bacteria and Archaea). All reads failing to pass filters (i.e. non-rRNA reads) were maintained as paired-ends reads and used in downstream analyses the command used for analysis is described in Additional file 2).

A subset of 150,000 single-end reads with length ≥ 80 nt and identified as bacterial 23S rRNA, the most predominant type of rRNA found in all samples, was uploaded in the SILVAngs data analysis service (https://www.arb-silva.de/ngs/) for identification of operational taxonomic units (OTUs).

### Assembly of NoV genomes

Non-rRNA paired-ends reads of samples OU1, OU2, OU3 and OU4 were mapped using Bowtie, version 2-2.2.5 [48] to Genbank reference sequences KF509946.2 (genotype GII.4 Sydney), KC631827.1 (genotype GII.4 2012 Sydney), KJ196277.1 (genotype GII.5) and JN899243.1 (genotype GI.7, partial genome), respectively, whereas SP1 and SP2 reads were aligned to KC576909 (genotype GII.4 2006b Den Haag). The consensus sequence of each sample was obtained from the alignments (SAM files) using Samtools.

Since no full-length or near full-length genome NoV GI.7 sequences were available in GenBank, additional steps were followed for OU4. OU4 reads were assembled *de novo* with Velvet version 1.2.10 using several hash lengths with read category set to ‘short paired’ and including the consensus sequence (partial ORF1, complete ORF2 and ORF3) as a long read (the command used for this analysis is described in Additional file 2). Norovirus contigs were identified among all Velvet assemblies with BLAST [49] using JN899243.1, a genotype GI.9 strain, as query sequence. All NoV contigs and the partial consensus sequence were aligned using MEGA 6.0 [50] to obtain the final genome assembly.

The ends of each NoV genome were extended beyond the reference sequences by using the first and last 15 nt as query for matches among fastq sequences using the Unix “grep” command. Matching reads were aligned to the consensus sequence and any extra nucleotides (5’or 3’ overhangs) were incorporated into the consensus sequence. The process was repeated until no more extra nucleotides were found at either end of the consensus sequence.

Sample reads were mapped with Bowtie 2 to their respective NoV extended consensus assembly. The final alignments (SAM files) were used to calculate the coverage per genome position using BEDTools, version 2.14.3-1 [51].

### NoV sequencing using Sanger’s method

The NoV strain from sample OU3 was sequenced using Sanger’s method to compare results against those obtained with Illumina MiSeq. Nine pairs of primers (described in Additional file 2) were designed to retrieve overlapping PCR amplicons between ~600 to 1100 bp long, spanning altogether all NoV ORFs. The RT and PCR reactions were performed as previously described [9]. PCR products were obtained for six out of nine pair of primers and were sequenced in both directions. The assembly of the sequences produced two non-overlapping contigs: a 3,448 bp sequence containing a partial ORF1 (incomplete at the 5’ and 3’ ends) and a 1,906 bp sequence spanning ORF2 and ORF3 (incomplete at the 5’ and 3’ ends, respectively).

### Characterization of non-rRNA, non-NoV sequences

The sequences failing to align with Bowtie 2 to the final NoV consensus sequence were analyzed with BLAST to further characterize the major components of stool RNA. All reads from a single end were queried against the non-redundant nt database using megablast (standalone version with databases downloaded on July 24, 2015; see parameters of BLAST analysis in Additional file 2). Results were analyzed with SPSS after removing duplicates, i.e. if a read had more than one BLAST hit, then only the hit with the lowest e-value was included in the analysis. Bacterial hits belonging to the normal human gut were identified using as reference the microoganisms reported in previous studies [52, 53].

### Analysis of single nucleotide variants

Single nucleotide variants (SNVs) were called with FreeBayes [54] and visually inspected in Tablet version 1.14.04.10 [55]. The following criteria was used for SNV calling with FreeBayes: -K (report all alleles passing filters), --haplotype_length =1 (call haplotypes as 1 nt long), –m or mapping quality =10 (chance that the read truly originated elsewhere of 1 in 10), –q or base quality =20 (chance of a wrong base call of 1 in 100), –F or alternate fraction =0.02 (call SNVs with frequencies ≥ 2 %), --min-coverage =10 (call SNVs for positions with coverage ≥10X) and –C or min-alternate-count = 5 (call SNVs with coverage ≥ 5X) (the command used for the analysis is described in Additional file 2). The choice to set up the analysis to detect variants with frequencies ≥ 2 % was made based on a study reporting that sequencing errors with the MiSeq platform can produce false variants that are undistinguishable from true low frequencies variants at ≤ 1 % with a 1000X average coverage [56]. We also set up the analysis to call SNVs with a coverage of ≥ 5X based on: 1) a study that eliminated virtually all false positives by calling variants if counted ≥ 10 times independently [57] and 2) the lower coverage per genome position achieved with our samples compared to Van den Hoecke et al. [57].

## Abbreviations

NoV, Norovirus; NGS, next generation sequencing; SSU, small subunit; LSU, large subunit; OTU, operational taxonomic unit; NRNNR, non-rRNA-non-NoV reads; SNV, single nucleotide variants; nsSNV, non-synonymous single nucleotide variants

## Additional files

Additional file 1: Major components of stool RNA after bacterial rRNA depletion. This file provides a description of the rRNA and non-NoV-non-rRNA reads from each sample. (DOCX 1108 kb) Additional file 2: Various supporting data. This file contains: 1) the data used to estimate the correlation between percentage of NoVreads and Ct values, 2) the commands used for NGS analysis, 3) a summary of the sequences filtered out by Prinseq-lite due to quality control and 4) a description of the primers designed for sequencing sample OU3 via Sanger's method. (DOCX 86 kb) Additional file 3: NoV SNV calling results. This file lists all synonymous and non-synonymous NoV SNVs detected by FreeBayes in samples SP1, SP2, and a subset of sequences from SP1 that produced an average NoV coverage similar to thatobserved in SP2. (XLSX 38 kb)

## References

[1] Ahmed SM, Hall AJ, Robinson AE, Verhoef L, Premkumar P, Parashar UD, Koopmans M, Lopman BA (2014). Global prevalence of norovirus in cases of gastroenteritis: a systematic review and meta-analysis. Lancet Infect Dis..

[2] Hardy ME (2005). Norovirus protein structure and function. FEMS Microbiol Lett..

[3] Vinje J (2015). Advances in laboratory methods for detection and typing of norovirus. J Clin Microbiol..

[4] Siebenga JJ, Vennema H, Zheng DP, Vinje J, Lee BE, Pang XL (2009). Norovirus illness is a global problem: emergence and spread of norovirus GII.4 variants, 2001-2007. J Infect Dis.

[5] Lindesmith LC, Donaldson EF, Lobue AD, Cannon JL, Zheng DP, Vinje J, Baric RS (2008). Mechanisms of GII.4 norovirus persistence in human populations. PLoS Med.

[6] Lindesmith LC, Beltramello M, Donaldson EF, Corti D, Swanstrom J, Debbink K, Lanzavecchia A, Baric RS (2012). Immunogenetic mechanisms driving norovirus GII.4 antigenic variation. PLoS Pathog..

[7] Lindesmith LC, Costantini V, Swanstrom J, Debbink K, Donaldson EF, Vinje J, Baric RS (2013). Emergence of a norovirus GII.4 strain correlates with changes in evolving blockade epitopes. J Virol.

[8] Eden JS, Tanaka MM, Boni MF, Rawlinson WD, White PA (2013). Recombination within the pandemic norovirus GII.4 lineage. J Virol.

[9] Hasing ME, Hazes B, Lee BE, Preiksaitis JK, Pang XL (2014). Detection and analysis of recombination in GII.4 norovirus strains causing gastroenteritis outbreaks in Alberta. Infect Genet Evol.

[10] Atmar RL, Opekun AR, Gilger MA, Estes MK, Crawford SE, Neill FH, Graham DY (2008). Norwalk virus shedding after experimental human infection. Emerg Infect Dis..

[11] Frange P, Touzot F, Debre M, Heritier S, Leruez-Ville M, Cros G, Rouzioux C, Blanche S, Fischer A, Avettand-Fenoel V (2012). Prevalence and clinical impact of norovirus fecal shedding in children with inherited immune deficiencies. J Infect Dis..

[12] Siebenga JJ, Beersma MF, Vennema H, van Biezen P, Hartwig NJ, Koopmans M (2008). High prevalence of prolonged norovirus shedding and illness among hospitalized patients: a model for in vivo molecular evolution. J Infect Dis..

[13] Ludwig A, Adams O, Laws HJ, Schroten H, Tenenbaum T (2008). Quantitative detection of norovirus excretion in pediatric patients with cancer and prolonged gastroenteritis and shedding of norovirus. J Med Virol..

[14] Schorn R, Hohne M, Meerbach A, Bossart W, Wuthrich RP, Schreier E, Muller NJ, Fehr T (2010). Chronic norovirus infection after kidney transplantation: molecular evidence for immune-driven viral evolution. Clin Infect Dis..

[15] Sukhrie FH, Siebenga JJ, Beersma MF, Koopmans M (2010). Chronic shedders as reservoir for nosocomial transmission of norovirus. J Clin Microbiol..

[16] Carlsson B, Lindberg AM, Rodriguez-Diaz J, Hedlund KO, Persson B, Svensson L (2009). Quasispecies dynamics and molecular evolution of human norovirus capsid P region during chronic infection. J Gen Virol..

[17] Karst SM, Baric RS (2015). What is the reservoir of emergent human norovirus strains?. J Virol..

[18] Hoffmann D, Hutzenthaler M, Seebach J, Panning M, Umgelter A, Menzel H, Protzer U, Metzler D (2012). Norovirus GII.4 and GII.7 capsid sequences undergo positive selection in chronically infected patients. Infect Genet Evol.

[19] Chan MC, Lee N, Ho WS, Law CO, Lau TC, Tsui SK, Sung JJ (2012). Covariation of major and minor viral capsid proteins in norovirus genogroup II genotype 4 strains. J Virol..

[20] Nilsson M, Hedlund KO, Thorhagen M, Larson G, Johansen K, Ekspong A, Svensson L (2003). Evolution of human calicivirus RNA in vivo: accumulation of mutations in the protruding P2 domain of the capsid leads to structural changes and possibly a new phenotype. J Virol..

[21] Bull RA, Eden JS, Luciani F, McElroy K, Rawlinson WD, White PA (2012). Contribution of intra- and interhost dynamics to norovirus evolution. J Virol..

[22] Vega E, Donaldson E, Huynh J, Barclay L, Lopman B, Baric R, Chen LF, Vinje J (2014). RNA populations in immunocompromised patients as reservoirs for novel norovirus variants. J Virol..

[23] Kundu S, Lockwood J, Depledge DP, Chaudhry Y, Aston A, Rao K, Hartley JC, Goodfellow I, Breuer J (2013). Next-generation whole genome sequencing identifies the direction of norovirus transmission in linked patients. Clin Infect Dis..

[24] Miyoshi T, Uchino K, Yoshida H, Motomura K, Takeda N, Matsuura Y, Tanaka T (2015). Long-term viral shedding and viral genome mutation in norovirus infection. J Med Virol..

[25] Batty EM, Wong TH, Trebes A, Argoud K, Attar M, Buck D (2013). A modified RNA-Seq approach for whole genome sequencing of RNA viruses from faecal and blood samples. PLoS One..

[26] Wong TH, Dearlove BL, Hedge J, Giess AP, Piazza P, Trebes A, Paul J, Smit E, Smith EG, Sutton JK, Wilcox MH, Dingle KE, Peto TE, Crook DW, Wilson DJ, Wyllie DH (2013). Whole genome sequencing and de novo assembly identifies Sydney-like variant noroviruses and recombinants during the winter 2012/2013 outbreak in England. Virol J.

[27] Cotten M, Petrova V, Phan MV, Rabaa MA, Watson SJ, Ong SH, Kellam P, Baker S (2014). Deep sequencing of norovirus genomes defines evolutionary patterns in an urban tropical setting. J Virol..

[28] Cotten M, Oude Munnink B, Canuti M, Deijs M, Watson SJ, Kellam P, van der Hoek L (2014). Full genome virus detection in fecal samples using sensitive nucleic acid preparation, deep sequencing, and a novel iterative sequence classification algorithm. PLoS One..

[29] de Vries M, Oude Munnink BB, Deijs M, Canuti M, Koekkoek SM, Molenkamp R, Bakker M, Jurriaans S, van Schaik BD, Luyf AC, Olabarriaga SD, van Kampen AH, van der Hoek L (2012). Performance of VIDISCA-454 in feces-suspensions and serum. Viruses..

[30] Bavelaar HH, Rahamat-Langendoen J, Niesters HG, Zoll J, Melchers WJ (2015). Whole genome sequencing of fecal samples as a tool for the diagnosis and genetic characterization of norovirus. J Clin Virol..

[31] He S, Wurtzel O, Singh K, Froula JL, Yilmaz S, Tringe SG, Wang Z, Chen F, Lindquist EA, Sorek R, Hugenholtz P (2010). Validation of two ribosomal RNA removal methods for microbial metatranscriptomics. Nat Methods..

[32] Giannoukos G, Ciulla DM, Huang K, Haas BJ, Izard J, Levin JZ, Livny J, Earl AM, Gevers D, Ward DV, Nusbaum C, Birren BW, Gnirke A (2012). Efficient and robust RNA-seq process for cultured bacteria and complex community transcriptomes. Genome Biol.

[33] Hasing ME, Lee BE, Preiksaitis JK, Tellier R, Honish L, Senthilselvan A, Pang XL (2013). Emergence of a new norovirus GII.4 variant and changes in the historical biennial pattern of norovirus outbreak activity in Alberta, Canada, from 2008 to 2013. J Clin Microbiol.

[34] Alam A, Qureshi SA, Vinje J, Zaidi A. Genetic characterization of norovirus strains in hospitalized children from Pakistan. J Med Virol. 2015; doi: 10.1002/jmv.24329 10.1002/jmv.24329 PMC5916762 26175018

[35] Debbink K, Lindesmith LC, Ferris MT, Swanstrom J, Beltramello M, Corti D, Lanzavecchia A, Baric RS (2014). Within-host evolution results in antigenically distinct GII.4 noroviruses. J Virol.

[36] Mai H, Gao Y, Cong X, Wang H, Liu N, Huang X, Xu L, Chen Y, Wei L.GII.4 Sydney 2012 norovirus infection in immunocompromised patients in Beijing and its rapid evolution in vivo. J Med Virol .2015; doi: 10.1002/jmv.24332. 10.1002/jmv.24332 26185038

[37] Bok K, Abente EJ, Realpe-Quintero M, Mitra T, Sosnovtsev SV, Kapikian AZ, Green KY (2009). Evolutionary dynamics of GII.4 noroviruses over a 34-year period. J Virol.

[38] Vongpunsawad S, Venkataram Prasad BV, Estes MK (2013). Norwalk Virus Minor Capsid Protein VP2 Associates within the VP1 Shell Domain. J Virol..

[39] Zhu S, Regev D, Watanabe M, Hickman D, Moussatche N, Jesus DM, Kahan SM, Napthine S, Brierley I, Hunter RN, Devabhaktuni D, Jones MK, Karst SM (2013). Identification of immune and viral correlates of norovirus protective immunity through comparative study of intra-cluster norovirus strains. PLoS Pathog.

[40] Bull RA, Luciani F, McElroy K, Gaudieri S, Pham ST, Chopra A, Cameron B, Maher L, Dore GJ, White PA, Lloyd AR (2011). Sequential bottlenecks drive viral evolution in early acute hepatitis C virus infection. PLoS Pathog..

[41] Ramachandran S, Campo DS, Dimitrova ZE, Xia GL, Purdy MA, Khudyakov YE (2011). Temporal variations in the hepatitis C virus intrahost population during chronic infection. J Virol..

[42] Shankarappa R, Margolick JB, Gange SJ, Rodrigo AG, Upchurch D, Farzadegan H, Gupta P, Rinaldo CR, Learn GH, He X, Huang XL, Mullins JI (1999). Consistent viral evolutionary changes associated with the progression of human immunodeficiency virus type 1 infection. J Virol..

[43] Mamanova L, Coffey AJ, Scott CE, Kozarewa I, Turner EH, Kumar A, Howard E, Shendure J, Turner DJ (2010). Target-enrichment strategies for next-generation sequencing. Nat Methods..

[44] Pang XL, Preiksaitis JK, Wong S, Li V, Lee BE (2010). Influence of novel norovirus GII.4 variants on gastroenteritis outbreak dynamics in Alberta and the Northern Territories, Canada between 2000 and 2008. PLoS One..

[45] Pang XL, Preiksaitis JK, Lee B (2005). Multiplex real time RT-PCR for the detection and quantitation of norovirus genogroups I and II in patients with acute gastroenteritis. J Clin Virol..

[46] Schmieder R, Edwards R (2011). Quality control and preprocessing of metagenomic datasets. Bioinformatics..

[47] Kopylova E, Noe L, Touzet H (2012). SortMeRNA: fast and accurate filtering of ribosomal RNAs in metatranscriptomic data. Bioinformatics..

[48] Langmead B, Salzberg SL (2012). Fast gapped-read alignment with Bowtie 2. Nat Methods..

[49] Altschul SF, Gish W, Miller W, Myers EW, Lipman DJ (1990). Basic local alignment search tool. J Mol Biol..

[50] Tamura K, Stecher G, Peterson D, Filipski A, Kumar S (2013). MEGA6: Molecular Evolutionary Genetics Analysis version 6.0.. Mol Biol Evol.

[51] Quinlan AR, Hall IM (2010). BEDTools: a flexible suite of utilities for comparing genomic features. Bioinformatics..

[52] Qin J, Li R, Raes J, Arumugam M, Burgdorf KS, Manichanh C (2010). A human gut microbial gene catalogue established by metagenomic sequencing. Nature..

[53] Walker AW, Ince J, Duncan SH, Webster LM, Holtrop G, Ze X (2011). Dominant and diet-responsive groups of bacteria within the human colonic microbiota. ISME J..

[54] Garrison E, Gabor M: Haplotype-based variant detection from short-read sequencing. 2012. arXiv preprint arXiv:1207.3907 [q-bio.GN]. Accessed 20 Jul 2015.

[55] Milne I, Bayer M, Cardle L, Shaw P, Stephen G, Wright F, Marshall D (2010). Tablet--next generation sequence assembly visualization. Bioinformatics..

[56] Thys K, Verhasselt P, Reumers J, Verbist BM, Maes B, Aerssens J (2015). Performance assessment of the Illumina massively parallel sequencing platform for deep sequencing analysis of viral minority variants. J Virol Methods..

[57] Van den Hoecke S, Verhelst J, Vuylsteke M, Saelens X (2015). Analysis of the genetic diversity of influenza A viruses using next-generation DNA sequencing. BMC Genomics.

