# Supplementary material for: A next generation sequencing-based method to study the intra-host genetic diversity of norovirus in patients with acute and chronic infection
Source: BMC Genomics. 2016 Jul 1;17:480. doi: 10.1186/s12864-016-2831-y (PMC4929757; doi:10.1186/s12864-016-2831-y)
Supplement: Additional file 1: — Major components of stool RNA after bacterial rRNA depletion. This file provides a description of the rRNA and non-NoV-non-rRNA reads from each sample. (DOCX 1108 kb) [file 12864_2016_2831_MOESM1_ESM.docx]

**Supplementary data 1:**

**Major components of stool RNA after bacterial rRNA depletion**

**1. Ribosomal RNA**

To determine whether our bacterial rRNA depletion process was effective we characterized all rRNA reads present in Illumina MiSeq NGS data from total (n=1) and bacterial rRNA-depleted (n=5) stool RNA. Bacterial 23S and 16S rRNA dominated the pool of total RNA with a combined abundance in the non-enriched OU4 sample reaching 99%. In the rRNA-depleted samples (OU1, OU2, OU3, SP1 and SP2) bacterial 23S and 16S rRNA remained prominently present with a combined abundance of 63.52% to 95.23%.

Table S1. Percentage of rRNA reads per source. rRNA reads were identified with SortmeRNA using the SILVA 119 small (16S/18S, SSU) and large subunit (23S/28S, LSU) and the rfam 5S and 5.8S ribosomal RNA databases for all three domains of life (*Bacteria*, *Archaea* and *Eukarya*).

|  | OU1 | OU2 | OU3 | OU4* | SP1 | SP2 |
| --- | --- | --- | --- | --- | --- | --- |
| Total reads | 7370512 | 4703034 | 9386008 | 8536368 | 5891654 | 2909390 |
| Reads passing QC filters | 7362154 (99.89%) | 4696680 (99.86%) | 9375772 (99.89%) | 8516490 (99.77%) | 5781324 (98.13%) | 2874654 (98.81%) |
| Per source**: |  |  |  |  |  |  |
| rRNA reads | 6429166 (87.33%) | 3054402 (65.03%) | 7651316 (81.61%) | 8461361 (99.35%) | 4591394 (79.42%) | 2751629 (95.72%) |
| Eukarya 28S | 4.75% | 0.70% | 5.83% | 0.01% | 0.54% | 0.21% |
| Eukarya 18S | 2.76% | 0.66% | 3.62% | 0.00% | 0.12% | 0.23% |
| Bacteria 23S | 70.87% | 57.01% | 60.87% | 73.82% | 75.30% | 90.71% |
| Bacteria 16S | 6.07% | 6.51% | 9.76% | 25.50% | 3.45% | 4.52% |
| Archaea 23S | 2.09% | 0.01% | 0.79 | 0.00% | 0.00% | 0.00% |
| Archaea 16S | 0.18% | 0.00% | 0.19% | 0.00% | 0.00% | 0.04% |
| 5S | 0.08% | 0.12% | 0.37% | 0.00% | 0.01% | 0.00% |
| 5.8S | 0.52% | 0.02% | 0.16% | 0.02% | 0.00% | 0.00% |
| non-rRNA reads | 932988 (12.67%) | 1642278 (34.97%) | 1724456 (18.39%) | 55129 (0.65%) | 1189930 (20.58%) | 123025 (4.28%) |

*Total stool (undepleted) RNA

**Percentages per source were calculated in reference to reads passing QC filters.

Although it is not possible to quantify the enrichment effect of rRNA depletion without paired samples, the percentages of NoV sequences obtained for each sample (Table 2) suggests that the non-rRNA fraction was enriched. For the non-enriched OU4 sample, complete elimination of rRNA would have given a 120-fold enrichment of NoV sequences (2.43% vs. 0.02%). Sample OU2, had the best (lowest) experimental enrichment factor, only 3-fold below the optimal (5.36% vs. 1.88%) whereas sample SP2 had the poorest (highest) experimental enrichment factor, 23-fold below the optimal (7.55% vs. 0.33%) but still considerably better than the non-enriched sample.

We noted that the best and worst experimental enrichment corresponded, respectively, to the samples with the lowest and highest remaining bacterial 23S rRNA fraction. In general, bacterial rRNA depletion seemed to perform better for 16S rRNA than for 23S rRNA. As a result, the ratio of 23S to 16S bacterial rRNA for depleted RNA samples was higher (6.4 to 21.8) compared to total RNA (2.9).

Further characterization of bacterial 23S rRNA reads showed a higher genetic diversity (i.e. higher number of OTUs for a given number of analysed sequences) in total RNA (OU4) vs. rRNA depleted RNA (OU1, OU2, OU3, SP1 and SP2) (Figure S1a). This suggests that the rRNA depletion process was biased towards removing sequences from some bacterial species or taxonomic groups.

Figure S1. Taxonomic fingerprint of bacterial rRNA 23S sequences present in stool RNA of individuals with NoV infection. A. Rarefaction curves (number of OTUs detected per number of reads). B. Distribution of bacterial rRNA 23S sequences by phyla. The analysis was performed using the SILVA NGS data analysis service.


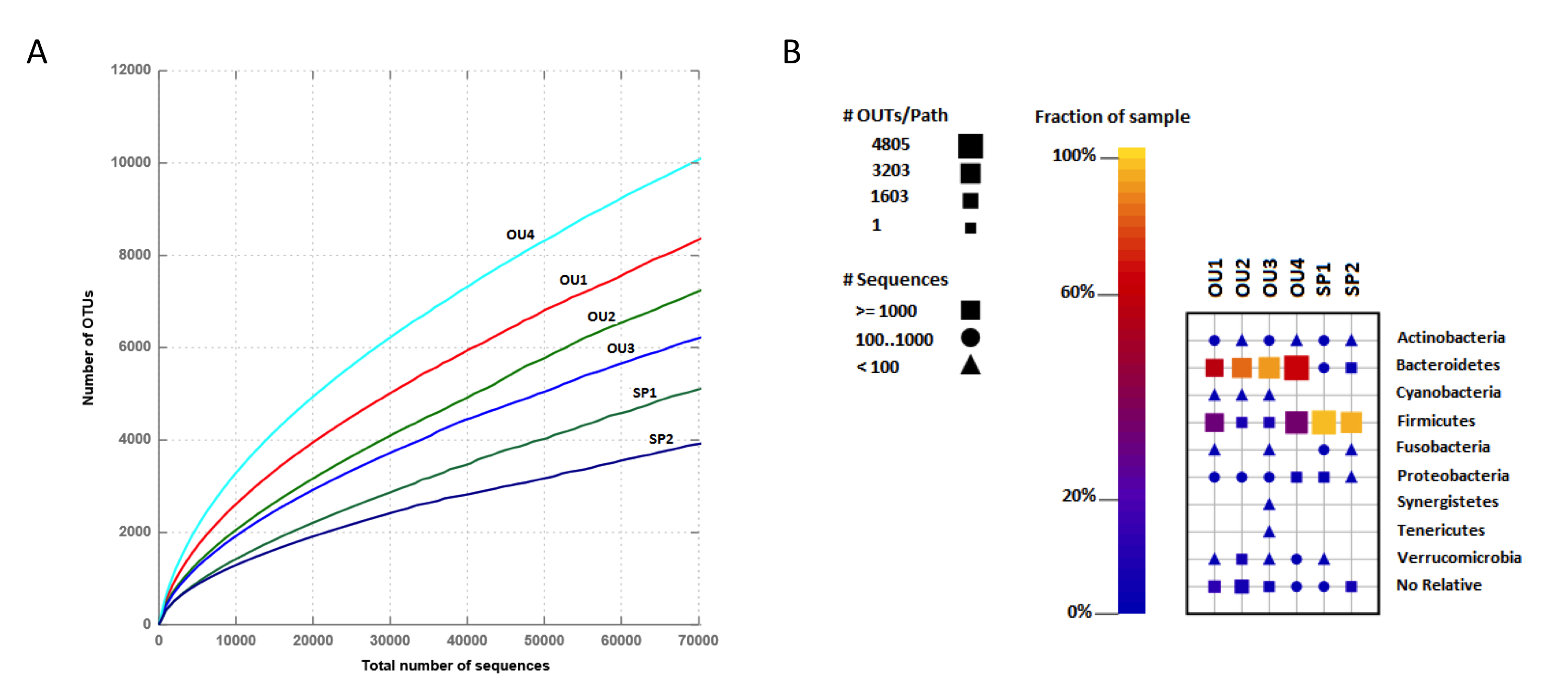


In all samples, the majority of bacterial 23S rRNA (94.5 to 99.9 %) was from the phyla Bacteroidetes and Firmicutes (Figure S1b). The lowest bacterial diversity was found in the samples from the immunocompromised patient, SP1 and SP2, which in contrast to the samples from outbreak patients, showed higher abundance of Firmicutes over Bacteroidetes. These relative abundances are in agreement with a previous study^[[1]](#footnote-1)^ describing that microbiome of subjects with NoV infections resembles that of healthy subjects, being dominated by the phylum Bacteroidetes with fewer members of Firmicutes; however, cases showing an increased number of Firmicutes over Bacteroidetes (as observed in our immunocompromised patient) have also been reported^1,^^[[2]](#footnote-2)^.

**2. Non-rRNA-non-NoV RNA**

In order to fully characterize other components of stool RNA, all non-rRNA-non-NoV reads were analyzed with BLAST. Between 45% and 90% of the non-rRNA-non-NoV had a BLAST hit (Figure S2). The kingdoms with most hits varied from sample to sample and were either Bacteria (OU1, OU2, OU3 and SP1) or Eukarya (OU4 and SP2).

The majority of bacterial hits (47-91%) were from microorganisms considered normal flora of the human gut. The bacterial species with most BLAST hits varied among samples and were: *Bacteroides fragilis* (40% and 18% of NRNNR in SP1 and OU2, respectively), *Faecalibacterium prausnitzii* (8% and 7% of NRNNR in OU1 and OU3), *Anaerostipes hadrus* (28% of the NRNNR in SP2) and an uncultured bacterium (3% of the NRNNR in OU4).

Eukaryotic hits were more abundant than bacterial hits in samples SP2 and OU4. Most of the eukaryotic hits in SP2 were from animal species considered common food sources (e.g. *Sus scrofa* and *Gallus gallus*). Almost all eukaryotic hits for OU4 (99%) were from *Xanthophyllomyces dendrorhous*, a yeast not yet reported to be part of the normal eukaryotic gut flora in humans in previous studies^[[3]](#footnote-3),^^[[4]](#footnote-4)^. *X. dendrorhous* was also identified in the other five samples although in lower proportion (3% to 20% in of NRNNR). Human sequences were found in all six samples representing 2.5%, 0.2%, 0.2%, 0.1%, 13.7% and 8.4% of NRNNR in OU1, OU2, OU3, OU4, SP1 and SP2, respectively.

Archaeal hits represented 0.01% to 1.5% of NRNNR among the six samples while viral hits were 0.05% to 0.8% of NRNNR. Viral hits included plant viruses and bacteriophages. Pepper Mild Mottle virus (PMMV), a plant virus previously identified in human stools^[[5]](#footnote-5)^, was present in samples OU1, OU2, SP1 and SP2. OU2 had PMMV sequences in sufficient amount to yield a full-length genome (GenBank accession number KU311159) with an average coverage of 27X. All six samples had a small proportion of NoV sequences that were missed and therefore not filtered by Bowtie (0.002% to 0.5% of NRNNR) as well as sequences from phage phiX174 (0.01% to 0.6% of NRNNR). The presence of the phiX174 phage was expected since a library of this phage was used as a spike-in control for the MiSeq sequencing run.

**Figure S2. Characterization of non-rRNA-non-NoV RNA in stool RNA.** The non-rRNA-non-NoV reads from six different stool samples were characterized using BLAST. Results were summarized at the kingdom level.


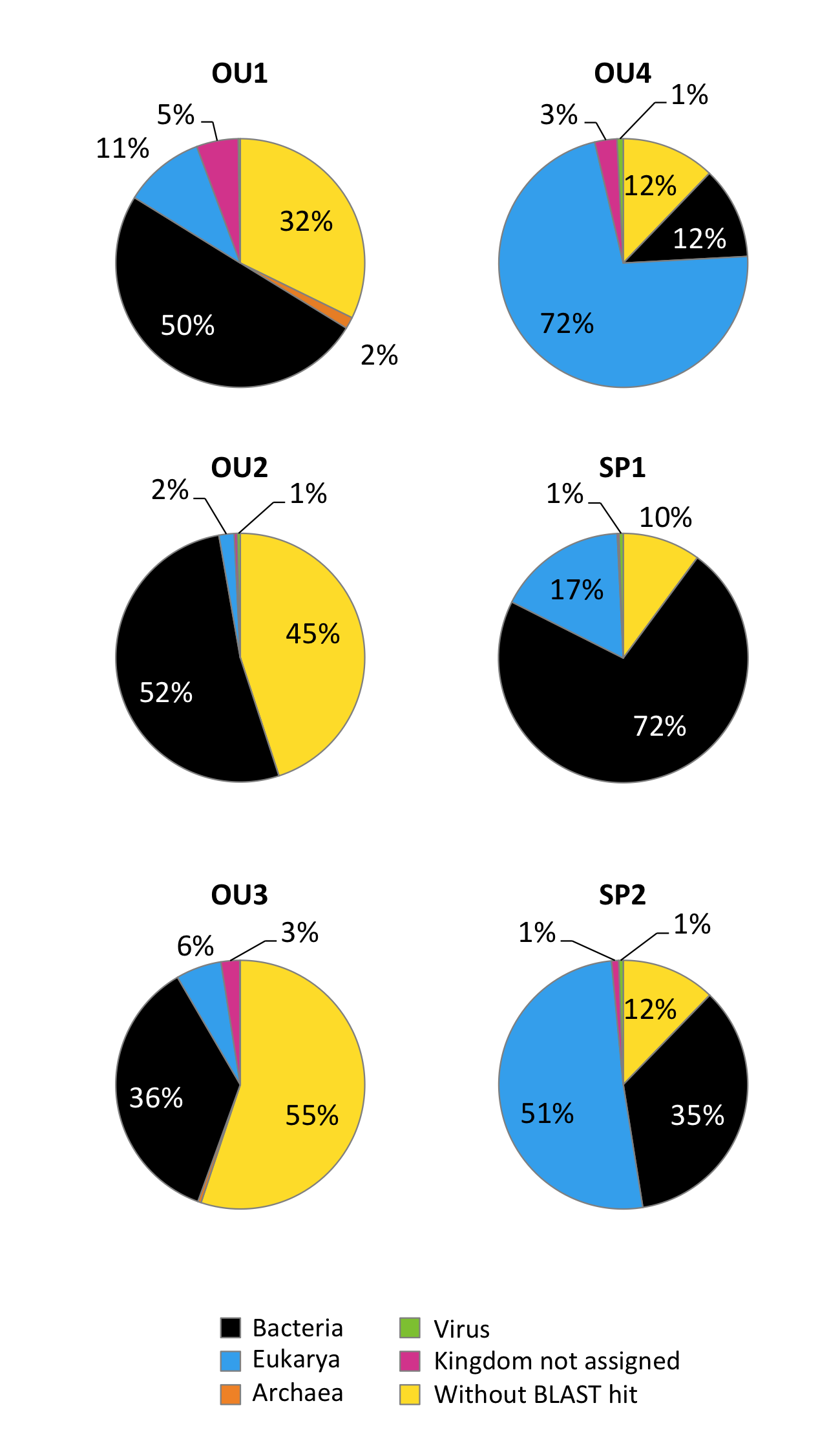


1. Nelson *et al.* PLoS One. 2012;7(10):e48224 [↑](#footnote-ref-1)
2. Youmans *et al.* Gut Microbes. 2015;6(2):110-9 [↑](#footnote-ref-2)
3. Scalan and Marchesi. ISME J. 2008 Dec;2(12):1183-93. [↑](#footnote-ref-3)
4. Hamad *et al* PLoS One. 2012;7(7):e40888 [↑](#footnote-ref-4)
5. Zhang *et al.* PLoS Biol. 2006 Jan;4(1):e3. [↑](#footnote-ref-5)
