# Supplementary material for: A next generation sequencing-based method to study the intra-host genetic diversity of norovirus in patients with acute and chronic infection
Source: BMC Genomics. 2016 Jul 1;17:480. doi: 10.1186/s12864-016-2831-y (PMC4929757; doi:10.1186/s12864-016-2831-y)
Supplement: Additional file 2: — Various supporting data. This file contains: 1) the data used to estimate the correlation between percentage of NoVreads and Ct values, 2) the commands used for NGS analysis, 3) a summary of the sequences filtered out by Prinseq-lite due to quality control and 4) a description of the primers designed for sequencing sample OU3 via Sanger's method. (DOCX 86 kb) [file 12864_2016_2831_MOESM2_ESM.docx]

**Supplementary data 2**

***Spearman’s rho correlation coefficient for % NoV reads***

| **Parameter** | **Spearman’s rho correlation coefficient** | ***P* (two-tailed)** |
| --- | --- | --- |
| Ct value | -0.886 | 0.019* |
| NoV copies per ng of RNA | 0.886 | 0.019* |
| NoV copies per uL of RNA | 0.886 | 0.019* |
| RNA concentration | 0.086 | 0.872 |

**Significant at the 0.05 level*

Data used for the analysis:

| Sample | % NoV reads | Ct value | NoV copies  (per uL of RNA) | RNA concentration by Nanodrop  (ng/uL) | NoV copies  (per ng of RNA) |
| --- | --- | --- | --- | --- | --- |
| OU1 | 0.51 | 20.58 | 484,264 | 129.48 | 3,740.1 |
| OU2 | 1.88 | 19.21 | 1,162,504 | 142.72 | 8,145.3 |
| OU3 | 0.01 | 28.90 | 2,376 | 161.88 | 14.7 |
| OU4 | 0.02 | 22.70 | 124,904 | 86 | 1,452.4 |
| SP1 | 1.78 | 21.92 | 205,632 | 97.88 | 2,100.9 |
| SP2 | 0.33 | 26.72 | 9,560 | 93.12 | 102.7 |

***Commands used for NGS data analysis***

Quality filtering and trimming or MiSeq sequence data using Prinseq-lite:

---------------------------------------------------------------------------------------------

$ perl prinseq-lite.pl -fastq **file1.fastq** -fastq2 **file2.fastq** -trim_qual_right 20 -trim_qual_left 20 -trim_qual_type min -trim_qual_rule lt -trim_qual_window 1 -trim_qual_step 1 -seq_id **file_IDs** -seq_id_mappings -graph_data **file_data** -graph_stats ld,gc,qd,ns,pt,ts -log **file_log** -min_qual_mean 20 -ns_max_p 90 -trim_left 1 -trim_right 1 -out_good **file_good** -out_bad **file_bad**

Filtering rRNA reads with SortMeRNA:

-------------------------------------------------

1) to merge paired-end reads:

$ bash merge-paired-reads.sh **file_1.fastq** **file_2.fastq file_1_2.fastq**

2) to filter reads using all rRNA databases:

$ sortmerna --ref silva-euk-28s-id98.fasta,silva-euk-28s-id98-db:silva-euk-18s-id95.fasta,silva-euk-18s-id95-db:silva-bac-23s-id98.fasta,silva-bac-23s-id98-db:silva-bac-16s-id90.fasta,silva-bac-16s-id90-db:silva-arc-23s-id98.fasta,silva-arc-23s-id98-db:silva-arc-16s-id95.fasta,silva-arc-16s-id95-db:rfam-5s-database-id98.fasta,rfam-5s-database-id98-db:rfam-5.8s-database-id98.fasta,rfam-5.8s-database-id98-db --reads **file_1_2.fastq** --aligned **file_1_2.fastq_rRNA.fastq** --other **file_1_2.fastq_norRNA.fastq** fastq --log --paired_out

3) to separate paired-end reads:

$ bash unmerge-paired-reads.sh **file_1_2.fastq_norRNA.fastq** **file_1_norRNA.fastq file_2_norRNA.fastq**

*De novo* assembly of Alberta404/2012 with Velvet:

-----------------------------------------------------------------

2) to run velveth for multiple hash lengths (15 to 75 with steps of 4) and a long reference sequence

$ velveth **15_75_longcontig**/ 15,75,4 -fastq -shortPaired **file_1_norRNA.fastq file_2_norRNA.fastq** -long **reference_sequence.fastq**

3) to run velvetg for each hash length specified in step 2:

$ velvetg **15_75_longcontig/_15** -ins_length **226** -ins_length_sd **102** -exp_cov **5**

Creating alignments of NoV with Bowtie:

----------------------------------------------------

1) to index reference sequence:

$ bowtie2-build **reference_sequence.fasta reference_sequence**

3) to create alignments (SAM files):

$ bowtie2 --local -x **reference_sequence** -1 **file_1_norRNA.fastq -2 file_2_norRNA.fastq** --un-conc **file_norRNAnoNoV_%** -S **file_NoV.sam**

Calculating coverage per site with Bedtools:

--------------------------------------------------------

1) to prepare a sorted BAM file from a SAM file:

$ samtools view -Sb **file_NoV.sam** > **file_NoV.bam**

$ samtools sort **file_NoV.bam file_NoV_sorted.bam**

2) to prepare a genome file:

created a text file with the information as follows: <chromName><TAB><chromSize>. For example:

OU1 7532

3) to run beedtools:

$ bedtools genomecov -d -ibam **file_NoV_sorted.bam** –g **genome_file** > **output_file_bedtools**

Analysis of SNPs with FreeBayes:

------------------------------------------

$ freebayes -f **reference_sequence.fasta** –K --haplotype-length 1 --min-alternate-fraction 0.02 -m 10 -q 20 --min-coverage 10 -C 5 --debug **file_NoV_sorted.bam** > **output_file_freebayes**

Characterization of non-rRNA-non-NoV reads with BLAST:

----------------------------------------------------------------------------

$ blastn -task megablast -db nt -query **file_norRNAnoNoV_1.fasta** -outfmt '6 qseqid qlen saccver evalue qlen length pident staxids sscinames scomnames sskingdoms' –best_hit_overhang 0.1 –best_hit_score_edge 0.1 -max_target_seqs 1 -out **blast_output_file**

***Prinseq-lite summary***

|  | **OU1** | **OU2** | **OU3** | **OU4** | **SP1** | **SP2** |
| --- | --- | --- | --- | --- | --- | --- |
| **Input sequences (file 1)** | 3,685,256 | 2,351,517 | 4,693,004 | 4,268,184 | 2,945,827 | 1,454,695 |
| **Input bases (file 1)** | 426,641,653 | 266,202,021 | 545,416,436 | 507,672,961 | 338,358,182 | 171,507,272 |
| **Input mean length (file 1)** | 115.77 | 113.20 | 116.22 | 118.94 | 114.86 | 117.90 |
| **Input sequences (file 2)** | 3,685,256 | 2,351,517 | 4,693,004 | 4,268,184 | 2,945,827 | 1,454,695 |
| **Input bases (file 2)** | 426,775,056 | 266,278,768 | 545,459,092 | 507,711,032 | 338,837,175 | 171,556,197 |
| **Input mean length (file 2)** | 115.81 | 113.24 | 116.23 | 118.95 | 115.02 | 117.93 |
| **Good sequences (pairs)** | 3,681,077 | 2,348,340 | 4,687,886 | 4,258,245 | 2,890,662 | 1,437,327 |
| **Good bases (pairs)** | 844,386,819 | 526,586,840 | 1,079,589,109 | 1,003,842,284 | 657,718,003 | 335,796,292 |
| **Good mean length (pairs)** | 229.39 | 224.24 | 230.29 | 235.74 | 227.53 | 233.63 |
| **Good sequences (singletons file 1)** | 3,759 (0.10%) | 2,953 (0.13%) | 4,367 (0.09%) | 9,106 (0.21%) | 54,719 (1.86%) | 16,772 (1.15%) |
| **Good bases (singletons file 1)** | 438,318 | 342,983 | 509,727 | 1,076,074 | 6,247,672 | 1,968,153 |
| **Good mean length (singletons file 1)** | 116.6 | 116.15 | 116.72 | 118.17 | 114.18 | 117.35 |
| **Good sequences (singletons file 2)** | 235 (0.01%) | 96 (0.00%) | 195 (0.00%) | 261 (0.01%) | 108 (0.00%) | 127 (0.01%) |
| **Good bases (singletons file 2)** | 27,051 | 11,100 | 22,608 | 30,488 | 12,389 | 14,675 |
| **Good mean length (singletons file 2)** | 115.11 | 115.62 | 115.94 | 116.81 | 114.71 | 115.55 |
| **Bad sequences (file 1)** | 420 (0.01%) | 224 (0.01%) | 751 (0.02%) | 833 (0.02%) | 446 (0.02%) | 596 (0.04%) |
| **Bad bases (file 1)** | 37,959 | 17,203 | 41,760 | 53,431 | 26,444 | 55,618 |
| **Bad mean length (file 1)** | 90.38 | 76.8 | 55.61 | 64.14 | 59.29 | 93.32 |
| **Bad sequences (file 2)** | 3,944 (0.11%) | 3,081 (0.13%) | 4,923 (0.10%) | 9,678 (0.23%) | 55,057 (1.87%) | 17,241 (1.19%) |
| **Bad bases (file 2)** | 462,920 | 361,209 | 546,244 | 1,119,542 | 6,601,351 | 2,061,632 |
| **Bad mean length (file 2)** | 117.37 | 117.24 | 110.96 | 115.68 | 119.9 | 119.58 |
| **Sequences filtered by trim_qual_left** | 276 | 223 | 1,110 | 1,088 | 623 | 368 |
| **Sequences filtered by min_qual_mean** | 4,088 | 3,082 | 4,564 | 9,423 | 54,880 | 17,469 |

***Primers used for sequencing NoV strain OU3 using Sanger’s***

| **Name** | **Type** | **Sequence** | **Start*** | **End*** | **Sense** | **Tm** | **Reference** |
| --- | --- | --- | --- | --- | --- | --- | --- |
| NVF1 | Forward | GTGAATGAAGATGGCGTCTA | -12 | 8 | + | 52.2 | Chhabra et al., Infect Genet Evol. 2010 Oct;10(7):1101-9 |
| 1GII5R | Reverse | AACTCCAAAGAGCTCTGCAAG | 950 | 970 | - | 55.3 | This study |
| 2GII5F | Forward | AGGATCTCATAGGGAAGTTGAG | 855 | 876 | + | 53.3 | This study |
| 2GII5R | Reverse | GCGCATCAGTCACAGGGTTGCTCATTC | 1686 | 1712 | - | 55.6 | This study |
| 3GII5F | Forward | CTCAGGTGATCAGAGAGTGG | 1591 | 1610 | + | 54.2 | This study |
| 3GII5R | Reverse | CAGTGGCCTCCACTTGTTTTTC | 2570 | 2591 | - | 56.7 | This study |
| 4GII5F | Forward | AGTGAGATACTATGTTAAATGTGTTCAAGAG | 2446 | 2476 | + | 55 | This study |
| 4GII5R | Reverse | GCGGTGTGCACCCCTATAA | 3510 | 3528 | - | 57.5 | This study |
| 5GII5F | Forward | AATGGGTACCCACGCCAC | 3352 | 3369 | + | 58 | This study |
| 5GII5R | Reverse | TTCAGGTTCAGCTGAGAACCTAAC | 4382 | 4405 | - | 56.6 | This study |
| 6GII5F | Forward | AGAGTTGGCATGAACATGAATG | 4241 | 4262 | + | 53.6 | This study |
| 6GII5R | Reverse | ACAAAATTAGTTCTAATCCAGGGGTC | 5253 | 5278 | - | 54.9 | This study |
| G2SKF | Forward | CNTGGGAGGGCGATCGCAA | 5070 | 5088 | + | 61.7 | Kojima et al., J Virol Methods. 2002 Feb;100(1-2):107-14 |
| 7GII5R | Reverse | CCTGTTTGCTGGATTGCTTTCAC | 6133 | 6155 | - | 57.2 | This study |
| 8GII5F | Forward | GTGACAGGACAGGTCCCTAATG | 5976 | 5997 | + | 57 | This study |
| 8GII5R | Reverse | GCTGGTGGTCTTCATTGAACC | 7055 | 7075 | - | 56.3 | This study |
| 9GII5F | Forward | TGATATGATAGCAATCAAACAGGGAG | 6925 | 6950 | + | 54.8 | This study |
| 9GII5R | Reverse | GACTCCCCCTTCTTGCGAAG | 7478 | 7497 | - | 57.9 | This study |
